# Traumatic Dental Injuries: Clinical Case Presentation and a 10-Year Epidemiological Investigation in an Italian Dental Emergency Service

**DOI:** 10.1155/2021/8649663

**Published:** 2021-06-29

**Authors:** Alberto Murri Dello Diago, Luigi Generali, Roberto Apponi, Vittorio Colombini, Vittorio Checchi

**Affiliations:** Department of Surgery, Medicine, Dentistry and Morphological Sciences-Unit of Dentistry and Oral-Maxillo-Facial Surgery, University of Modena and Reggio Emilia, Modena, Italy

## Abstract

Traumatic dental injuries (TDIs) are very common in the world population, and international literature reports several studies which helped in the definition of international guidelines. The aim of this study is to present two clinical cases of TDI and to investigate epidemiological and etiological aspects of TDIs in patients treated in Modena, Italy, between January 2010 and December 2020. The presented case reports are two explicative clinical cases of successful TDI management with a long-term follow-up. The epidemiological analysis was performed on patients who visited the Dental Emergency Service of the Dentistry and Oral-Maxillo-Facial Surgery Unit of Modena (Italy) over a period of 10 years. Data relating to age, gender, type of trauma, and place of accident were collected. Five-hundred-sixty-five TDIs that occurred to patients from 1 to 68 years old were reported, with a total of 860 injured teeth. The peak age at which TDIs are most represented varies between 2 and 3 years old, and they occurred frequently from 1 up to 7 years old. 57.5% were male, while 42.5% were female. The most common trauma resulted to be the uncomplicated crown fracture (20%), immediately followed by lateral luxation (19%), intrusive luxation (18%), avulsion (17%), and complicated crown fracture (15%). TDIs occurred at home in 44% of cases. The need for more prevention training must be highlighted, due to the fact that many TDIs occur at home and in a preschool age.

## 1. Introduction

Traumatic dental injuries (TDIs) are common in the worldwide population. Although the oral cavity represents a small component of the human body, TDIs represent 5% of all health injuries and up to 17% in pediatric patients [1]. A recent study shows that more than one billion living people have had TDIs [1]. Scientific literature shows a great variability regarding the incidence and prevalence of TDIs in the world population [2–4]. Although the International Association of Dental Traumatology (IADT) had codified precise guidelines [5–7], this variability is related to several reasons, such as the diversity in TDI classification methods and parameter recording, and the different cultural and social contexts in which the various studies had been performed.

TDIs mainly affect the pediatric population, with a high percentage of preschool children and adolescents, and hardly concern elderly patients, if not in a small percentage [1]. The most frequent TDIs in patients with deciduous teeth are periodontal injuries and luxation, while hard tissue injuries and consequent crown fractures are more specific and related to patients with permanent dentition [7]. Trauma such as avulsion and complicated fractures determine functional and esthetic issues that could affect social relationships; numerous studies have shown how TDIs could lead to relationship difficulties and therefore be the reason for personal, domestic, and social issues [3, 8, 9].

The aim of this article is to present two explicative clinical cases of TDI management and to investigate TDI frequency, patterns, and causes in patients treated in Modena, Italy, between January 2010 and December 2020.

## 2. Case Presentation

Making use of a digital clinical chart archiving system, TDI cases that occurred at the Dental Emergency Service of the Dental Clinic of Modena University, between 1 January 2010 and 31 December 2020, were selected. All patients have been treated according to the protocols established by the IADT guidelines [5–7], enhanced with photographic documentation, radiographic examination, pulp status evaluation, and instrumental investigations. According to the principle of minimum invasiveness and with the aim of optimizing the oral health-related quality of life, individualized treatment plans for each patient were implemented in line with scientific evidence [10–14].

All the anamnestic questionnaires and the TDI-related information were collected, and the following data were obtained: demographic data (gender and age); causes of trauma (sports injuries, accidents at work, collisions, violence and abuse, unintentional falls, and road accidents); location of the trauma (home, school, leisure activities, sports environment, and work); type of trauma, classified according to IADT classification [5–7]; and teeth involved (permanent or deciduous, maxillary or mandibular, and anterior or posterior). All data were registered into Microsoft Excel version 14.1.0 for Macintosh.

Among all selected TDIs, the management of two explicative and challenging clinical cases is described, following CARE reporting guidelines for case reports.

### 2.1. Clinical Case #1

**Figure 1:**
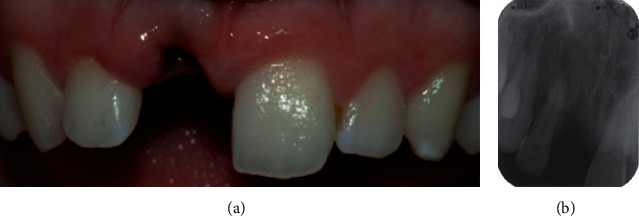
Posttraumatic (a) clinical and (b) radiographic view showing the absence of the upper right central incisor.

A 15-year-old female patient was referred due to traumatic avulsion of her right upper central incisor that occurred during a volleyball game (Figure 1).

**Figure 2:**
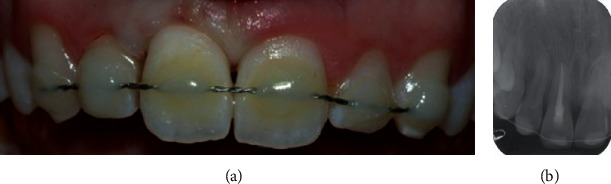
(a) Clinical and (b) radiographic view of the upper incisor after reimplantation and splint.

This tooth had been kept in an extraoral dry environment for two hours and had a mature apex, and for this reason, nonviable soft tissues were removed from the root with a gauze and endodontic therapy was carried out prior to replantation. After local anesthesia administration, the socket was irrigated with saline solution and carefully checked to exclude the presence of bony fractures. The tooth was then replanted applying slight but firm pressure. The correct position of the tooth was verified clinically and radiographically, and it was stabilized through a passive flexible splint made by a 0.4 mm diameter metal wire bonded to the tooth and to adjacent teeth (Figure 2).

**Figure 3:**
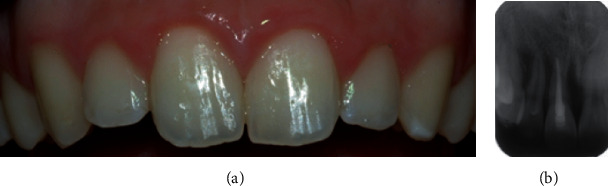
(a) Clinical and (b) radiographic view of the upper incisor at the 3-year follow-up visit.

Postoperative instructions included antibiotic therapy with amoxicillin, soft diet for 2 weeks, and soft-bristle toothbrush and chlorhexidine 0.12% mouth rinses, twice a day for 2 weeks. The splint was kept in place for 2 weeks, and the patient was visited after 2, 3, and 6 months and yearly. At the 3-year follow-up visit, the tooth appeared asymptomatic, with physiological mobility, no sensitivity to percussion, and normal percussion sound. No radiotransparency and no radiographic evidence of root resorption were detected (Figure 3).

**Figure 4:**
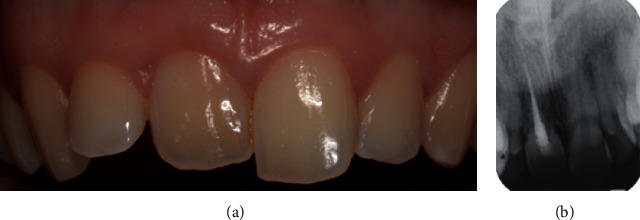
(a) Clinical and (b) radiographic view of the upper incisor at the 8-year follow-up visit: tooth infraposition and root resorption are evident.

At the 8-year follow-up visit instead, the tooth presented no mobility, metallic percussion sound, and clinical infraposition. Radiographically, there was evidence of ankylosis-related resorption (Figure 4).

### 2.2. Clinical Case #2

**Figure 5:**
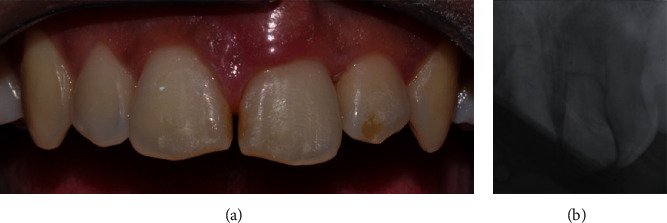
Posttraumatic (a) clinical and (b) radiographic view. Gingival tissues around the upper left central incisor are swollen and red, while a horizontal root fraction is detectable from the periapical X-ray.

A 20-year-old male patient was referred due to traumatic root fracture of his left upper central incisor after a bike accident (Figure 5).

A passive and flexible buccal splint with metal stainless steel wire and composite patches was immediately performed and kept for 4 weeks, aimed at stabilizing the mobile coronal segment and maintaining the vitality of the tooth. Soft diet was suggested for 1 week and oral hygiene instructions were delivered, brushing with a soft-bristle toothbrush and rinsing with chlorhexidine 0.12% mouthwash to prevent accumulation of plaque and biofilm.

**Figure 6:**
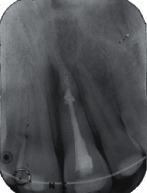
Radiographic view of the upper left central incisor after endodontic treatment.

However, after one month, the tooth still did not respond to electrometric and thermal pulp testing, due to plausible pulp necrosis development. For this reason, an endodontic treatment of the coronal tooth segment to the fracture line had to be performed (Figure 6).

**Figure 7:**
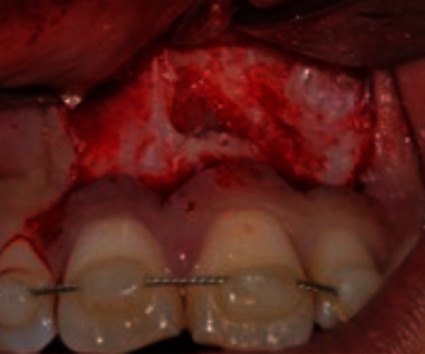
Surgical view of the upper left central incisor during apicectomy.

After a further 4 weeks, the patient still referred pain and discomfort of the area, and the decision for a surgical approach had to be taken. An apicectomy with a retrograde canal obturation was performed in order to remove the symptomatology and obtain a complete healing of the area (Figure 7).

Postsurgical instructions included prevention of further injury by avoidance of contact sports, meticulous oral hygiene, and rinsing with an antibacterial agent such as chlorhexidine gluconate 0.12%.

**Figure 8:**
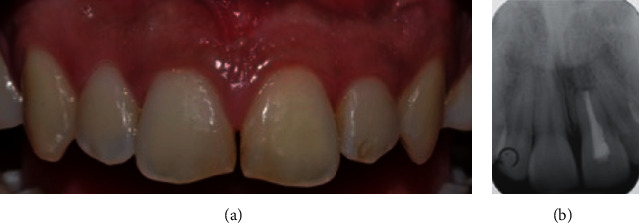
(a) Clinical and (b) radiographic view 4 years after the surgical therapy.

Healing was uneventful, and the patient did not refer pain nor swelling. At the 4-year follow-up visit, clinical and radiographic analysis showed a healed area with a good ossification of the periradicular bone (Figure 8).

### 2.3. Epidemiological Results

Out of a total of 26355 patients who accessed the Dental Emergency Service unit in the period from January 2010 to December 2020, 565 TDIs were registered. Patients' age varied from 0 to 68 years, and the peak age at which TDIs were most represented varied between 2 and 3 years old; however, it was highly represented from 1 up to 7 years old.

In 40% of the cases, only one tooth was involved, in 38% two teeth, in 15% three teeth, and in 7% four teeth, for a total of 860 injured teeth.

**Figure 9:**
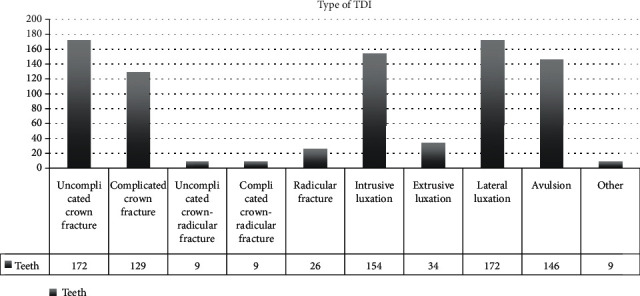
TDI frequency based on the type of injury.

The most frequent injury resulted in an uncomplicated crown fracture (20% of cases), immediately followed by lateral luxation. Successively, this was followed by 18% of intrusive luxation, 17% of avulsion, and 15% of complicated crown fracture (Figure 9). Crown-root fractures were represented in the 2% of the cases. The 57.5% of affected patients were male, while 42.5% were female. The most frequent TDI among males resulted in uncomplicated crown fracture, while the most frequent TDI among females resulted in lateral luxation.

TDIs involved permanent dentition in 63% of patients: maxillary anterior teeth involvement was observed in 55.5% of cases, mandibular anterior in 6.5%, while posterior teeth were involved in only 1% of the cases. The remaining 37% involved deciduous teeth.

**Figure 10:**
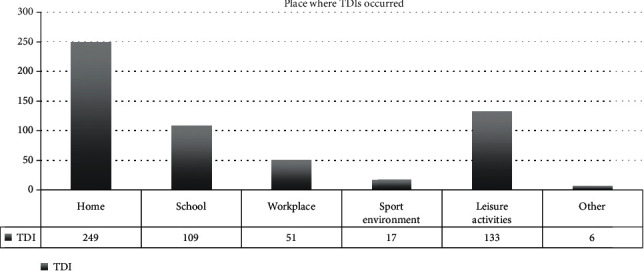
TDI frequency based on the place where injuries occurred.

TDIs occurred mainly at home (44%), followed by leisure activities (23.5%), school (19.5%), and workplace (9%) (Figure 10).

**Figure 11:**
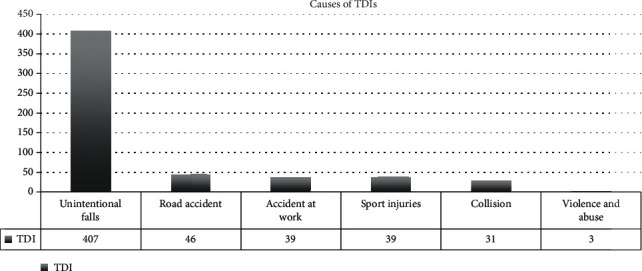
TDI frequency based on the cause of injury.

Trauma was caused by unintentional falls in 72% of cases, by road accidents in 8%, by accidents at work in 7%, by sports injuries in 7%, by consequences of collision in 5.5%, and by violence and abuse in the remaining 0.5% (Figure 11).

## 3. Discussion

In the present epidemiological analysis, avulsions were found in 17% of the total population. When related to deciduous teeth, these represented 11.6%, while in permanent teeth these represented 5.4%, in agreement with other authors [6, 15], but proportionally slightly higher than the percentage reported by Andreasen et al. (4%) [16].

In a recent comprehensive review, guidelines for the treatment of avulsed teeth were published [6]. In these cases, the choice of treatment is related to the maturity of the root (open or closed apex) and to the condition of the periodontal ligament (PDL) cells. The survival of these cells is dependent on the time out of the mouth and on the storage liquid in which the avulsed tooth is kept, since PDL cells are nonviable after an extraalveolar dry time of 30 minutes [6]. In Clinical Case #1, the total extraoral dry time had been more than 60 minutes, and therefore, the PDL cells were likely to be nonviable. Literature demonstrated that delayed replantation has a poor long-term prognosis and that the periodontal ligament becomes necrotic, is not expected to regenerate, and the expected outcome is ankylosis-related root resorption [6]. The goal of replantation in Clinical Case #1 was to restore, although temporarily, esthetics and function while maintaining alveolar bone height, width, and contour.

The possibility of replacing avulsed tooth in a growing patient with implant-prosthetic rehabilitations through mini-implants has proved to be an effective therapeutic option [17–22].

In this epidemiological analysis, luxation occurred in 41% of the total cases: 27.2% in deciduous teeth and 13.9% in permanent teeth. Lateral luxation represented 20% and intrusive luxation 18%, while extrusive luxation occurred only in 3% of the sample. These data represent a high frequency if compared with Andreasen et al.'s results who reported 9% frequency for nonspecific luxations [16]. Treatment usually involves tooth reposition and semiflexible splinting, as documented by the IADT guidelines [5–7].

Similarly to Andreasen et al.'s results [16], the present epidemiological analysis reported that complicated crown fractures occurred in 15% of the cases, showing a higher frequency than other studies [3, 23]. The most represented TDI was the uncomplicated crown fracture, observed in 20% of the cases, while other authors reported different frequencies, such as 13% [23], 86% [24], 94% [6], and 50% [16]. In these cases, a therapeutic approach with a direct composite reconstruction should be preferably performed.

In the present data collection, crown-root fractures were represented in 2% of the cases: 0.3% concerned deciduous teeth and 1.7% concerned permanent teeth. Root fractures were 3%: 0.2% concerned deciduous teeth and 2.8% concerned permanent teeth. The study by Andreasen et al. instead reports different results, such as 9% of crown-root fractures and 7% of root fractures [16]. The treatment of crown-root fractures depends on the level of the fracture along the root, and therefore different kinds of therapies could be addressed.

In accordance with Glendor et al., it was observed that in 55% of the cases, the involved teeth were permanent maxillary incisors, while in 6.5%, those involved were the permanent mandibular ones [3, 8]. Deciduous teeth were involved in 37% of the cases, according to Piovesan et al.'s results that reported rates between 9.4% and 41% [24, 25]. In 40% of the cases, only one tooth was involved; in 36%, two teeth were involved; and in 22%, more than two teeth were involved. This was different from Andreasen et al.'s study that reported a greater difference in cases involving one (80%) and two (18%) teeth [16].

A recent comprehensive review gave useful indications for the treatment of root fractures [5]. In detail, no endodontic treatment should be started at the emergency visit since pulp necrosis and infection could develop later and usually in the coronal fragment only [5]. If endodontic therapy is requested, treatment of the only coronal segment is indicated, and apexification may be needed if the determination of the root canal length may be challenging due to oblique fracture lines [5]. In Clinical Case #2, root canal therapy had to be performed after one month since the tooth did not respond to thermal pulp testing nor electrometric test. Following IADT guidelines, only the coronal part of the fractured tooth was treated. However, after another month, a surgical approach had to be chosen in order to solve the patient's symptomatology that was still present.

The results of this epidemiological analysis show that TDI frequency treated in the Dental Emergency Service of the Dentistry and Oral-Maxillo-Facial Surgery Unit of Modena, Italy, are in line with what is recorded in other national and international data.

Concerning a patient's age, the results of this epidemiological investigation are only partially in agreement with previous researches that, although with a larger population sample, revealed a TDI average age incidence of about 15 years [3, 8]. In our case, the peak diagnosis of TDIs has been observed between 2 and 3 years old. The mobility skills of a child between 2 and 3 years of age are still to be developed since they must still acquire an adult confidence. However, the desire to overcome personal limits often pushes the child to test himself, thus risking his own safety, and these aspects could explain the TDI peak incidence in this age group. Other possible causes, such as lack of supervision or child abuse, have to be considered.

In accordance with previous studies [3, 8, 9], about 46% of TDIs were however found in the age group between 1 and 7 years (preschool). At this stage of life, children are very dynamic and sports activities are increasingly practiced, leading to TDIs being consequently much more frequent.

As demonstrated by several studies [3, 8, 9], a prevalence of TDI has been observed among male subjects (57.5%), rather than female (42.5%). This prevalence appears equal to the findings of Glendor et al.'s results that show a 2 : 1 ratio of TDIs between males and females [7].

Concerning TDI reasons, it emerged that the main cause was an unintentional fall, especially in preschool age, which most frequently caused luxation and avulsion. Domestic falls and accidental impacts against objects, in particular interior furnishings such as tables, chairs, and sinks, were recorded in 72% of total cases.

Road accidents, which range from falling from a bicycle or moped to, more rarely, car accidents, were reported in 8% of the cases. According to these data, accidents at work are also frequent along with sports injuries, causing a significant incidence of avulsions. Different types of TDIs can be observed based on the type of sport activity and therefore of the impact dynamic. High-speed sports seem to lead to bone fractures, while low-speed sports lead to dental injuries. These data are in agreement with previous studies on the etiology of trauma [22, 26–33]. In fact, sports injuries have been observed in patients aged 12 to 32, road accidents have been observed in patients aged 17 to 62, and workplace accidents have been observed only in adult patients, as well as collisions. In a similar way, in a recent study, the majority of TDI patients were adolescents (27.9%), or younger than 10 years (23.2%). The main cause of TDI was cycling (43.5%), followed by sports such as football and baseball [34].

Although there are well-coded international prevention guidelines, TDIs are still very relevant in pediatric patients. Therefore, there is a need for the development of training and prevention programs for TDI, organizing adequate emergency services, and planning awareness campaigns.

## Data Availability

Data supporting the conclusions of the study can be accessed by asking the corresponding author.
